# Medication-Related Osteonecrosis of the Jaw Spontaneously Occurred in a Patient with Chronic Myelogenous Leukemia Only by Imatinib: A Report of a Rare Case

**DOI:** 10.1155/2021/6621937

**Published:** 2021-01-27

**Authors:** Makiko Okubo-Sato, Kenji Yamagata, Satoshi Fukuzawa, Kazuhiro Terada, Fumihiko Uchida, Naomi Ishibashi-Kanno, Hiroki Bukawa

**Affiliations:** Department of Oral and Maxillofacial Surgery, Institute of Clinical Medicine, Faculty of Medicine, University of Tsukuba, 1-1-1 Tennodai, Tsukuba, Ibaraki 305-8575, Japan

## Abstract

The prevalence of medication-related osteonecrosis of the jaw (MRONJ) associated with molecular-targeted therapies such as bevacizumab and sunitinib has been constantly increasing in recent years. MRONJ frequently occurs after invasive dental procedures such as tooth extraction in patients currently or with a previous history of receiving antiresorptive agents including bisphosphonates and/or denosumab. Here, we report a rare case of spontaneously occurring MRONJ of the mandible in a 52-year-old Japanese woman with chronic myelogenous leukemia (CML) who was administered imatinib for 9 years. She had never been treated with antiresorptive agents, and her MRONJ developed spontaneously. Although there have been few reports of MRONJ related to imatinib, our case reported here indicates that imatinib may be capable of causing spontaneous MRONJ.

## 1. Introduction

Medication-related osteonecrosis of the jaw (MRONJ) is a severe adverse drug effect related to antiresorptive agents, and the number of reports of MRONJ cases associated with molecular-targeted therapies including antibody drugs and tyrosine kinase inhibitors (TKIs) has been increasing [1, 2]. Most cases are related to the administration of antiresorptive medications, such as bisphosphonates and receptor activator of nuclear factor *κ*B ligand (RANKL) inhibitors, and precipitating events, such as tooth extraction, which may significantly increase the risk of developing MRONJ. Meanwhile, frequently reported agents among molecular-targeted therapies associated with MRONJ are bevacizumab [3, 4], sunitinib [5], sorafenib [6], everolimus [7], temsirolimus [3], and aflibercept [8].

Imatinib mesylate (Glivec®) is a TKI with specificity for the BCR–ABL protein resulting from the t(9, 22)-derived Philadelphia chromosome, platelet-derived growth factor receptors (PDGFRs), and c-kit. It is administered in patients with chronic myelogenous leukemia (CML), acute lymphocytic leukemia, and c-kit-positive gastrointestinal stromal tumor (GIST) [9]. The documented adverse effects of imatinib are nausea, vomiting, edema, tumor necrosis, muscle cramps, hematologic side effects, cardiovascular side effects, hepatic side effects, nephrotoxicity, and dermatologic side effects (such as skin rashes, pruritus, and petechiae); however, little is known about MRONJ [10]. The present report describes a very rare case of spontaneous MRONJ related to only imatinib in the absence of antiresorptive agents.

## 2. Case Report

**Figure 1:**
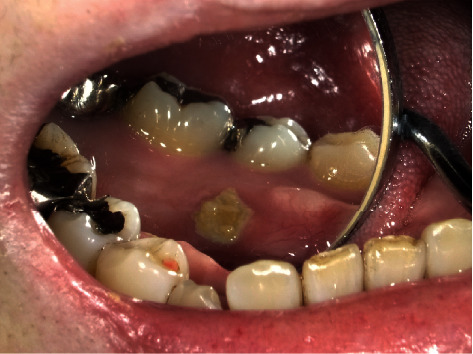
Examination of the oral cavity. Intraoral examination revealed bone exposure measuring approximately 5 mm on the lingual surface of the mandibular tori around the second premolar.

**Figure 2:**
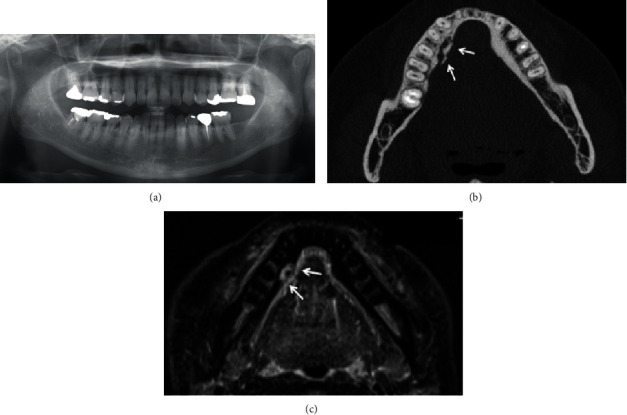
Imaging findings. (a) Panoramic radiography showed no obvious bone destruction or marginal and apical periodontitis. (b) Axial computed tomography depicted a sequestrum measuring approximately 25 mm (arrow). (c) Axial magnetic resonance short T1 inversion recovery imaging showed abnormal intramedullary signal intensity (arrow) on the lingual side of the right lower premolar.

**Figure 3:**
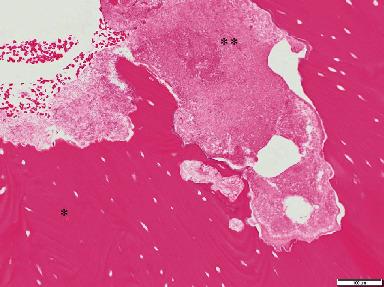
Microscopic findings. Histopathological examination revealed necrotic lamellar bone with empty lacunae (asterisk) and bacterial aggregates (double asterisk) (hematoxylin and eosin staining ×200 magnification).

**Figure 4:**
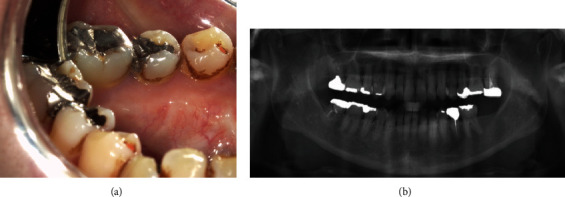
Follow-up oral examination 2 years after the surgery. (a) Follow-up observation revealed complete soft tissue healing with no signs of refractory osteonecrosis and exposed bone. (b) Panoramic radiography was also clear.

A 52-year-old Japanese woman was referred to our department in July 2018. She complained of exposed bone with contact pain in the right mandible that had persisted for 6 months. She started using an oral antimicrobial mouthrinse, and the exposed bone was eliminated several times by her general practitioner, but her condition did not improve. She was diagnosed with CML in 2008 and administered only imatinib (400 mg daily) continuously with no apparent side effects. There was no other past medical history, and she had never received antiresorptive agents or undergone radiotherapy in the head and neck region. Clinical examination showed that the exposed bone measured approximately 5 mm on the lingual surface of the mandibular tori around the right premolars (Figure 1). Purulent discharge, swelling, tooth mobility, and percussion pain were not observed, and her oral hygiene was favorable. Although there were no significant findings on panoramic radiography (Figure 2(a)), computed tomography showed a separated sequestrum measuring approximately 25 mm, and magnetic resonance imaging indicated abnormal intramedullary signal intensity in the right mandible (Figures 2(b) and 2(c)). She was clinically diagnosed with MRONJ stage II. In November 2018, sequestrectomy and removal of the mandibular tori were performed under general anesthesia because of her vomiting reflex. Histopathological examination revealed necrotic lamellar bone with empty lacunae, bacterial aggregates, and an absence of neoplastic cells (Figure 3). Her postoperative course was uneventful, and at the 2-year follow-up, she was stable with no signs of MRONJ recurrence (Figures 4(a) and 4(b)).

## 3. Discussion

In 2014, the American Association of Oral and Maxillofacial Surgeons (AAOMS) suggested that some cases of osteonecrosis of the jaw have been observed in patients with cancer following treatment with angiogenic inhibitors (bevacizumab), TKIs (sunitinib, sorafenib), and mammalian target of rapamycin (mTOR) inhibitors (sirolimus). The committee remains concerned about a similar potential risk associated with several other medications within the same drug class that have a similar mechanism of action [11]. Bevacizumab, sunitinib, and sorafenib inhibit vascular endothelial growth factor- (VEGF-) mediated angiogenesis and ultimately delay wound healing, which play relevant roles in the onset of MRONJ. Inhibition of mTOR, VEGF expression, and angiogenesis are also depressed [12].

Imatinib inhibits PDGFR and c-kit, which are both related to angiogenesis, and some reports have indicated that imatinib may partly reduce VEGF expression through the inhibition of these factors [13, 14]. Apart from its vascular effects, imatinib directly affects bone remodeling. The inhibition of c-fms and c-kit signaling by imatinib may decrease osteoclast number and activity. Imatinib also inhibits the proton-generating activity of carbonic anhydrase II and suppresses bone resorption [15]. In addition, the inhibition of PDGFR signaling in osteoblasts inhibits the production of macrophage colony-stimulating factor and RANKL, which are essential for osteoclast genesis, and decreases osteoblast proliferation [16]. Therefore, imatinib treatment should be included as a potential risk factor for developing MRONJ because of its inhibitory effects on angiogenesis and bone remodeling. Our patient had never received antiresorptive agents and spontaneously developed MRONJ, as suggested by the aforementioned mechanism.

There are only three reports describing MRONJ related to imatinib (Table 1). Nicolatou-Galitis et al. [17] reported a case of MRONJ that developed after tooth extraction in a woman with CML receiving imatinib for 19 months. Four years prior, she had received chemotherapy and rituximab for non-Hodgkin's lymphoma, as well as alendronate and one injection of zoledronate for osteoporosis. MRONJ was treated but recurred following an injection of denosumab. Mohamed et al. [18] reported a patient with chronic lymphocytic leukemia (CLL) treated with imatinib for 13 months. The patient was administered alendronate for 119 months, followed by denosumab for 5 months until 5 months before tooth extraction, which was a trigger for MRONJ. The last case reported by Viviano et al. [19] involved a patient with MRONJ after 22 months of receiving imatinib for a GIST. Although the patient had never received antiresorptive agents, a tooth was extracted prior to the patient developing MRONJ. Two of the three reported cases of MRONJ due to imatinib involved the administration of antiresorptive agents. Our case is the second case of MRONJ caused only by imatinib and is the first case of spontaneous development without any surgical invasion. Although the exposed bone was on the mandibular tori, which is usually considered an anatomical risk factor of mechanical stimulation, the patient had no clear history of trauma involved in the pathogenesis, such as wearing dentures and chewing hard foods. AAOMS (2014) recommended conservative treatments for patients with stage II MRONJ. However, the symptom of some patients with stage II and III do not respond to conservative treatments and reported that surgical management recently had a good prognosis [19]. The Position Paper 2017 of the Japanese Allied Committee on Osteonecrosis of the Jaw also recommended surgical treatments for stage II ONJ [20]. In our case, the patient continued to use antimicrobial mouthrinse but her condition did not improve. Therefore, we selected surgical treatment.

To the best of our knowledge, we present the first case of spontaneous MRONJ attributed to the chronic use of imatinib with no history of receiving antiresorptive agents. The dental and medical communities must be aware that even if there are no apparent risk factors of MRONJ, the condition may develop in patients treated only with imatinib, and long-term monitoring and care are required.

## Figures and Tables

**Table 1:** Reported MRONJ cases related to imatinib [17–19].

No.	Study (yr)	Age (yr)/gender	Disease	Period of imatinib treatment (mo)	Antiresorptive agent	Trigger event	Stage	Treatment	Clinical outcome
1	Nicolatou-Galitis et al. (2013) [17]	71/M	CML	19	Alendronate Zoledoronate Denosumab	Tooth extraction	II	Antibiotic	Improved
2	Viviano et al. (2017) [19]	72/M	GIST	22	None	Tooth extraction	II	Antibiotic	Unknown
3	Mohamed et al. (2018) [18]	81/F	CLL	13	Alendronate Denosumab	Tooth extraction	II	Surgery	Healed
4	Present case (2020)	52/F	CML	111	None	None	II	Surgery	Healed

Abbreviations: MRONJ: medication-related osteonecrosis of the jaw; F: female; M: male; CML: chronic myelogenous leukemia; GIST: gastrointestinal stromal tumor; CLL: chronic lymphocytic leukemia.
